# RNA m6A detection using raw current signals and basecalling errors from Nanopore direct RNA sequencing reads

**DOI:** 10.1093/bioinformatics/btae375

**Published:** 2024-06-18

**Authors:** Peng Ni, Jinrui Xu, Zeyu Zhong, Feng Luo, Jianxin Wang

**Affiliations:** School of Computer Science and Engineering, Central South University, Changsha 410083, China; Xiangjiang Laboratory, Changsha 410205, China; Hunan Provincial Key Lab on Bioinformatics, Central South University, Changsha 410083, China; School of Computer Science and Engineering, Central South University, Changsha 410083, China; Xiangjiang Laboratory, Changsha 410205, China; Hunan Provincial Key Lab on Bioinformatics, Central South University, Changsha 410083, China; School of Computer Science and Engineering, Central South University, Changsha 410083, China; Xiangjiang Laboratory, Changsha 410205, China; Hunan Provincial Key Lab on Bioinformatics, Central South University, Changsha 410083, China; School of Computing, Clemson University, Clemson, SC 29634-0974, United States; School of Computer Science and Engineering, Central South University, Changsha 410083, China; Xiangjiang Laboratory, Changsha 410205, China; Hunan Provincial Key Lab on Bioinformatics, Central South University, Changsha 410083, China

## Abstract

**Motivation:**

Nanopore direct RNA sequencing (DRS) enables the detection of RNA N6-methyladenosine (m6A) without extra laboratory techniques. A number of supervised or comparative approaches have been developed to identify m6A from Nanopore DRS reads. However, existing methods typically utilize either statistical features of the current signals or basecalling-error features, ignoring the richer information of the raw signals of DRS reads.

**Results:**

Here, we propose RedNano, a deep-learning method designed to detect m6A from Nanopore DRS reads by utilizing both raw signals and basecalling errors. RedNano processes the raw-signal feature and basecalling-error feature through residual networks. We validated the effectiveness of RedNano using synthesized, *Arabidopsis*, and human DRS data. The results demonstrate that RedNano surpasses existing methods by achieving higher area under the ROC curve (AUC) and area under the precision-recall curve (AUPRs) in all three datasets. Furthermore, RedNano performs better in cross-species validation, demonstrating its robustness. Additionally, when detecting m6A from an independent dataset of *Populus trichocarpa*, RedNano achieves the highest AUC and AUPR, which are 3.8%–9.9% and 5.5%–13.8% higher than other methods, respectively.

**Availability and implementation:**

The source code of RedNano is freely available at https://github.com/Derryxu/RedNano.

## 1 Introduction

Currently, over 170 chemical modifications have been found in RNA nucleotides (Jonkhout *et al.* 2017, Boccaletto and Bagiński 2021). As one of the most abundant RNA modifications, N6-methyladenosine (m6A) exists in approximately 25% mRNAs (Meyer and Jaffrey 2014). In eukaryotic RNA, m6A occurs mostly in the DRACH or RRACH motif (where D = A, G, or U, R = A or G, H = A, C or U). m6A impacts mRNA structure (Liu *et al.* 2015) and stability (Wang *et al.* 2014) and plays an important role in multiple biological processes, such as mRNA translation (Wang *et al.* 2015), pre-mRNA splicing (Louloupi *et al.* 2018), and primary microRNA processing (Alarcón *et al.* 2015).

Experimental methods that rely on immunoprecipitation or chemoselective alteration, such as MeRIP-seq (Meyer *et al.* 2012), miCLIP (Linder *et al.* 2015), and m6ACE-Seq (Koh *et al.* 2019), are currently the gold standard for the detection of RNA m6A (Leger *et al.* 2021). However, these methods have several limitations, such as lack of single-nucleotide resolution, isoform ambiguity, and biases induced by the complex experimental steps (Leger *et al.* 2021). Recently, Zhang *et al.* (2021) incorporated a modification-free RNA library into these experimental methods as a negative control, which reduced abundant false positives induced by sequencing bias or RNA structure. Hu *et al.* (2022) proposed m6A-SAC-Seq that uses dimethyltransferase MjDim1 to label m6A. Liu *et al.* (2023) proposed GLORI, an m6A quantification method that uses glyoxal and nitrite-mediated deamination of unmethylated adenosines while keeping m6A intact. Both m6A-SAC-Seq and GLORI can quantify m6A modification at single-nucleotide resolution in the mammalian transcriptome. However, these two methods still need complex experimental protocols during the library preparation procedure.

The Nanopore direct RNA sequencing (DRS) technology offers new opportunities for the detection of RNA m6A (Lucas and Novoa 2023). When an RNA molecule passes through a Nanopore, different nucleotides in the RNA produce distinctive current signals. Modifications in nucleotides also influence the current signals. Recently, a number of comparative methods and supervised methods have been developed for identifying m6A modification from Nanopore DRS reads (Zhong *et al.* 2023). These methods rely on either the signal differences or the basecalling errors in the Nanopore reads introduced by base modifications (Jain *et al.* 2022). Comparative methods, including DRUMMER (Price *et al.* 2020), ELIGOS (Jenjaroenpun *et al.* 2021), Yanocomp (Parker *et al.* 2021), Nanocompore (Leger *et al.* 2021), and xPore (Pratanwanich *et al.* 2021), identify m6A sites by applying statistical tests to compare the differences between two groups of samples: one is the sample where the m6A sites to be identified, another is a m6A-free sample as a control. Comparative methods do not require pre-training. However, control samples such as METTL3 knock-out (KO) cells are sometimes difficult to generate (Hendra *et al.* 2022) and also bring extra costs.

Supervised methods, including EpiNano (Liu *et al.* 2019), MINES (Lorenz *et al.* 2020), nanom6A (Gao *et al.* 2021), m6anet (Hendra *et al.* 2022), and DENA (Qin *et al.* 2022), use machine learning to identity m6A sites from Nanopore DRS reads. Supervised methods require labeled sites (m6A and non-m6A sites) to train the machine learning models. However, supervised methods do not require any control samples for the detection. Among these five representative methods, EpiNano utilizes systematic basecalling errors as features (Liu *et al.* 2019), while the other four methods all generate features from the current signals of Nanopore reads. Epinano trains a support vector machine model by utilizing base quality, mismatch frequency, and deletion frequency of the targeted base (or k-mer) as features. The model of EpiNano was trained using DRS reads of in-vitro synthetic transcripts. MINES first generates the fraction-modified values of Tombo (Stoiber *et al.* 2017) from the current signals of Nanopore reads, then applies a random forest classifier to detect m6A sites from human HEK293T and HeLa cell lines (Lorenz *et al.* 2020). nanom6A trains an extreme gradient boosting model by utilizing the mean, median, standard deviation, and the number of the current signals of each base as features (Gao *et al.* 2021). m6Anet trains a multiple-instance learning framework using DRS data of human HEK293T and HCT116 cell lines (Hendra *et al.* 2022). The features that m6Anet uses include the sequence around the targeted base, and the normalized mean, standard deviation, and the dwelling time of the raw signals of each 5-mer in the sequence. DENA trains an authentic neural network model using the mean, median, standard deviation, dwell time, and base quality for each base in a 5-mer (Qin *et al.* 2022). The performance of DENA for detecting and quantifying m6A was validated using the DRS data of *Arabidopsis*.

Supervised methods still have limitations. First, most of the currently supervised methods employ either the statistical features of the current signals or the basecalling-error features, which do not utilize both types of features for RNA modification detection. Second, these methods ignore raw current signals with indeterminate length, which contain sequential time information that statistical features do not have. The raw current signal values have not yet been used for m6A detection. In addition, supervised methods are effective for detecting RNA modifications in specific motifs (RRACH/DRACH) of the trained species, but the capacity of these methods is inadequate when migrating the pre-trained models to a different species.

In this study, we present a supervised method, called RedNano, for the detection of RNA m6A from Nanopore DRS reads. RedNano utilizes deep neural networks to process the features from raw electrical signals and basecalling errors of Nanopore reads. We evaluate RedNano on multiple datasets, including synthesized (Liu *et al.* 2019), *Arabidopsis* (Parker *et al.* 2020), and human (Pratanwanich *et al.* 2021) DRS data. The results show that RedNano achieves higher AUCs and area under the precision-recall curve (AUPRs) than other methods. Additionally, RedNano exhibits robust performance when applying the pre-trained models to different species. Moreover, we trained a multi-species model of RedNano using the training data from the three species, which showed an overall superior performance compared to the models trained with single-species data. The experiment on *Populus trichocarpa* data also validates the effectiveness of the multi-species model.

## 2 Materials and methods

### 2.1 Data

#### 2.1.1 Nanopore sequencing data

We first used three groups of DRS datasets for training and testing. The three datasets contain DRS reads of the synthesized RNA, *Arabidopsis*, and human, respectively. For the synthesized RNA dataset, there are two biological replicates for each “curlcake” experiment condition (un-modified RNA and m6A-modified RNA), which can be accessed in the National Center for Biotechnology Information (NCBI) under accession PRJNA511582 (Liu *et al.* 2019). For *Arabidopsis*, we got the DRS reads of vir-1, and VIR-complemented (VIRc) lines through the European Nucleotide Archive (ENA) under accession PRJEB32782 (Parker *et al.* 2020). For humans, we used the DRS reads of wild-type HEK293T cells, which were obtained from the ENA under accession PRJEB40872 (Pratanwanich *et al.* 2021). In addition, we used the DRS reads of *P.trichocarpa* as an independent datset for testing, which were obtained from the NCBI SRA accession SRR8491764 and SRR1267667527 (Gao *et al.* 2021). Details of all used DRS datasets are provided in Supplementary Table S1.

#### 2.1.2 References and m6A ground truth

**(1) Reference sequences**. The reference sequences of the synthesized RNA were downloaded from Liu *et al.* (2019), including four synthesized RNA sequences with respective lengths of 2276, 2492, 2625, and 2742. For humans, we downloaded GRCh38 Ensembl release version 91 as the reference genome/transcriptome. For *Arabidopsis*, we got the TAIR10 reference and gene annotation from NCBI (Wheeler *et al.* 2007) and Araport11 (Cheng *et al.* 2017), respectively. For *P.trichocarpa*, we used the *P.trichocarpa* v3.0 assembly and the corresponding gene annotation from the Joint Genome Institute (JGI) as the reference genome/transcriptome (Tuskan *et al.* 2006).

**(2) m6A ground truth**. For the synthesized RNA dataset, we use all RRACH/DRACH sites in un-modified and m6A-modified Nanopore reads as the benchmark. For *Arabidopsis*, we got 3106 m6A sites from Parker *et al.* (2020). We consider these sites in the DRS data of the VIRc and vir-1 mutant as modified and unmodified sites, respectively. For humans, m6A sites of the HEK293T cell line are obtained by using m6ACE-seq (Koh *et al.* 2019) and miCLIP (Linder *et al.* 2015). We include the sites that are METTL3-dependent (WT/KO relative methylation level ratio ≥4.0, *P* value of one-tailed *t*-test <.05) from the m6ACE-seq data as the m6A sites (Pratanwanich *et al.* 2021). We also include the m6A sites of miCLIP from Linder *et al.* (2015). In total, we got 20 725 DRACH m6A sites (including 17 702 RRACH sites) and considered any other DRACH site in the human genome as unmodified. For *P.trichocarpa*, we performed m6A prediction from the MeRIP-seq data from Gao *et al.* (2021) by using the MeRIPseqPipe pipeline (Bao *et al.* 2022). We got 122 075 RRACH m6A sites in total (Note S1).

### 2.2 RedNano

**Figure 1.**
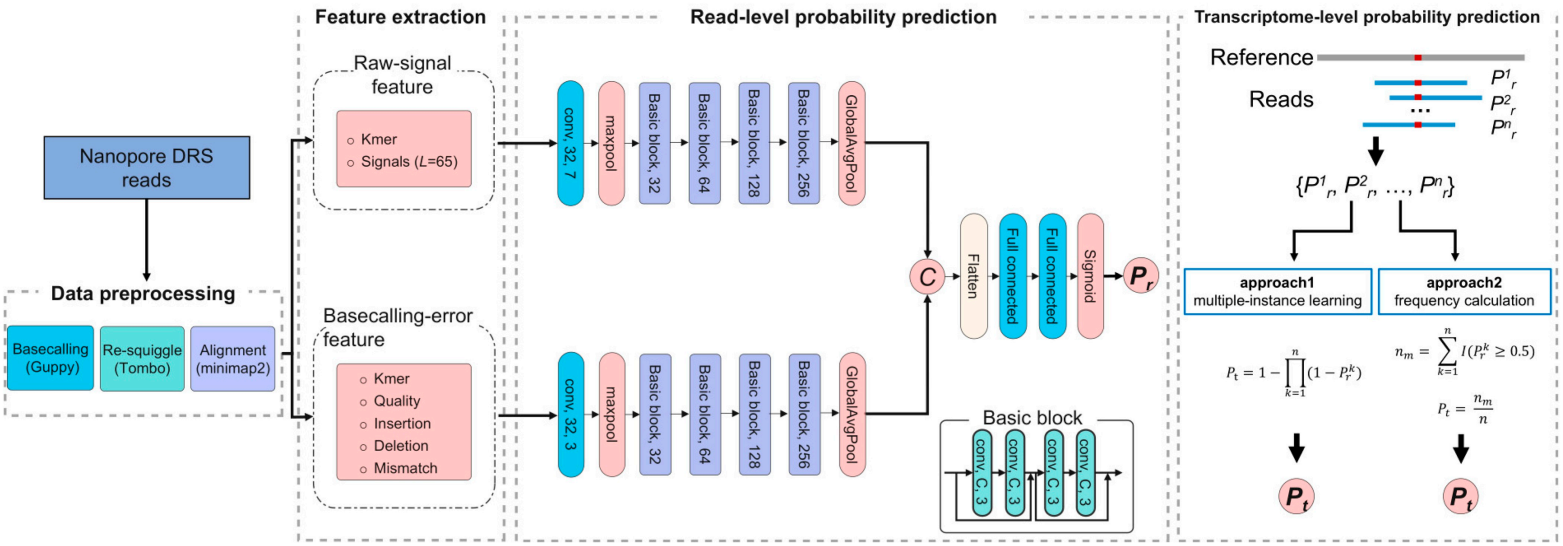
Pipeline of RedNano for detecting RNA m6A from Nanopore DRS reads. *L* is the fixed signal length; (conv, 32, 7) represents a 1D convolution layer with 64 output channels and a kernel size of 7; $P_{r}$ is the read-level methylation probability; $P_{t}$ is the trascriptome-level methylation probability.

RedNano utilizes both raw-signal features and basecalling-error features from Nanopore DRS reads to detect m6A sites (Fig. 1). In the pipeline of RedNano, the Nanopore DRS reads are first preprocessed for feature extraction. Then, RedNano extracts the raw signals and the basecalling characteristics of the targeted sites from the reads as the features of the deep-learning model. RedNano applies residual networks to process the two types of features. Then, RedNano outputs the read-level methylation probability of each targeted site. By aggregating the read-level predictions, RedNano calculates the transcriptome-level methylation probability of the targeted site. The details of the pipeline of RedNano are described below.

#### 2.2.1 Data preprocessing

Several steps must be taken prior to extracting features from Nanopore DRS reads. First, the raw Nanopore DRS reads in FAST5 format are basecalled using the official basecaller of Oxford Nanopore Technologies (Wick *et al.* 2019), Guppy (v3.1.5), to generate the nucleotide sequence of each read. Second, we use minimap2 (v2.17) (Li 2018) to align each read to the reference transcriptome. We also map the raw current signals of each read to the contiguous bases in the reference transcriptome with the re-squiggle module of Tombo (v1.5.1) (Stoiber *et al.* 2017). After the re-squiggle step, we can get the corresponding signals of each matched base in the transcriptome. Downstream manipulations (such as sorting) of Sequence Alignment/Map files (in SAM/BAM format) are performed by Samtools (version 1.7) (Li *et al.* 2009). The commands and parameters of each tool used for data preprocessing are provided in Note S2.

#### 2.2.2 Feature extraction

For each targeted base A (in DRACH/RRACH motif) in a read, RedNano extracts the 5-mer sequence centered on it to generate features. Different from other methods, RedNano uses both the raw-signal feature and basecalling-error feature from Nanopore DRS reads for m6A detection. The raw-signal feature of each targeted site is extracted from the re-squiggled signal-to-sequence alignment of the 5-mer. The basecalling-error feature is generated from the read-to-reference sequence alignment of the 5-mer. The details of the two types of features are described below.

1. Raw-signal feature. After the re-squiggle step, we first use the median shift and median absolute deviation scale to normalize the raw current signals of each read (Stoiber *et al.* 2017, Ni *et al.* 2019). As the matched current signals of each base are variable in length, we randomly select *L* normalized signal values of each base as the input to the following deep-learning model. By statistically counting the number of signals corresponding to each base in the synthesized DRS data, we set *L* to 65 (Supplementary Fig. S1). If the number of signals of a base is <65, we pad with zeros. We use one-hot embedding to encode the nucleotide sequence of each 5-mer. To match the sequence of the normalized signals, we expand the embedding vector of each base *L* times (*L* = 65). At last, we form a 5*325 matrix as the raw-signal feature for a targeted site in a read (Supplementary Fig. S2A).
2. Basecalling-error feature. To get the basecalling-error feature of a targeted site, we first covert the read-to-reference sequence alignments into tab-delimited format with the *sam2tsv* module of jvarkit (https://github.com/lindenb/jvarkit). Then for the 5-mer of the targeted site, we extract the status (match, mismatch, deletion, insertion) and the base quality of each base in the 5-mer from the tab-delimited file. At last, by combining the basecalling characteristics and the embedding vectors of the 5-mer, we form an 8*5 matrix as the basecalling-error feature of the targeted site in a read (Supplementary Fig. S2B).

#### 2.2.3 Model architecture of RedNano

In RedNano, we use deep residual networks (ResNet) (He *et al.* 2016) to process the two different types of features separately (Fig. 1). Both ResNets for the two types of features comprise four basic residual blocks. Each basic residual block contains four convolutional layers with identical output channel numbers and kernel size. The output channels for the convolutional layers in the four basic residual blocks are 32, 64, 128, and 256, respectively. Each convolutional layer in the basic blocks is followed by a batch normalization layer and an ReLU activation function. The output from the final basic block undergoes processing by a global average pooling layer. For the raw-signal features, the ResNet is preceded by a 1D CNN layer (with 32 output channels and a kernel size of 7) and a maxpooling layer. The ResNet for the basecalling-error features is also preceded by a 1D CNN layer (with 32 output channels and a kernel size of 3) and a maxpooling layer.

The processed raw-signal features and basecalling-error features are ultimately concatenated and flattened into a 1D array. This 1D array is then fed into a fully connected neural network comprising two hidden layers. The output from this network is processed by a sigmoid activation function, generating the final prediction score $P_{r}\in$[0,1] of a targeted site in a read as follows:

(1) $$P_{r}=\frac{1}{1+e^{-x}}$$

where *x* is the output of the fully connected neural network. The prediction score *P_r_* generated by RedNano represents the methylation probability of a targeted site at the read level.

RedNano utilizes two approaches to calculate the transcriptome-level methylation probability *P_t_* by grouping the predictions of a targeted site in the aligned reads (Fig. 1). In the first approach, RedNano uses multiple-instance learning (Hendra *et al.* 2022) as follows:

(2) $$P_{t}=1-\prod_{k=1}^{n}\left(1-p_{r}^{k}\right)$$

where $p_{r}^{k}$ is the read-level methylation probability of the targeted site in the *k*-th read, *n* represents the total number of reads. As m6Anet (Hendra *et al.* 2022) does, RedNano always samples 20 reads for each prediction of *P_t_*. In the second approach, RedNano calculates *P_t_* of a targeted site as the methylation frequency as follows:

(3) $$n_{m}=\sum_{k=1}^{n}I(p_{r}^{k}\geq 0.5)$$

(4) $$P_{t}=\frac{n_{m}}{n}$$

where *n_m_* represents the number of read-level methylation probabilities that are ≥.5.

We train and test RedNano using independent DRS reads. Specifically, we use different technical replicates in each dataset to train and test RedNano (Supplementary Table S1). Details of model training and testing of RedNano are provided in Supplementary Note S3 and Fig. S3.

## 3 Results

### 3.1 Evaluation of RedNano for RNA m6A detection on three DRS datasets

We assess RedNano in detecting RNA m6A using three DRS datasets, including the synthesized RNA dataset, the *Arabidopsis* dataset, and the human HEK293T dataset (Supplementary Table S1). We compare RedNano with four other supervised methods: EpiNano (v1.2), nanom6A (v2.0), DENA (commit b888322), and m6Anet (v1.0). As shown in Supplementary Table S2, these four methods vary in terms of targeted motifs and the data used for training. To ensure fairness, we first train and test the models of RedNano using each of these three datasets independently. For each dataset, we compare corresponding methods that were trained using the dataset of the same species (Supplementary Tables S1 and S2). All methods compared were executed using their default parameters. We test both approaches of RedNano for predicting transcriptome-level methylation probabilities using all three datasets (Supplementary Table S3). According to the results, we chose the model trained using the multiple-instance learning approach on the human dataset, and the models trained using the frequency calculation approach on the synthesized RNA and *Arabidopsis* datasets to compare with other methods.

**Figure 2.**
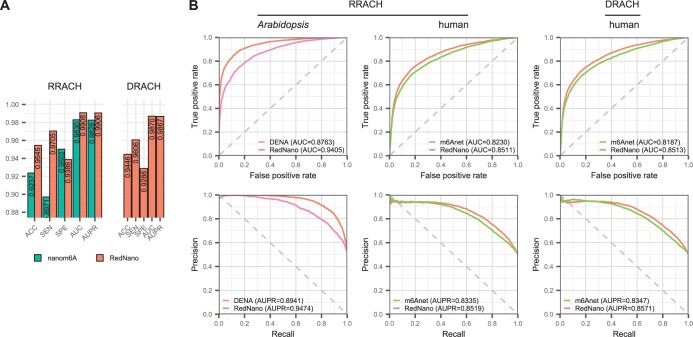
Performance comparison of RedNano and other methods using three DRS datasets independently. (A) Performance comparison of RedNano and nanom6A at read level using the synthesized RNA dataset. (B) Performance comparison of RedNano and other methods in detecting m6A sites at transcriptome level using the *Arabidopsis* and human datasets.

First, we compare RedNano and nanom6A at the read level using the synthesized RNA dataset. Although EpiNano is also trained using the synthesized RNA dataset, it does not provide predictions at the read level. Hence, we exclude it. As shown in Fig. 2A, for detecting RNA m6As in the RRACH motif, RedNano outperforms nanom6A on all evaluation criteria: accuracy (ACC), sensitivity (SEN), specificity (SPE), area under the ROC curve (AUC), and AUPR. Notably, RedNano gets *a* > 0.95 accuracy and *a* > 0.99 AUC. Additionally, we assess RedNano on the DRACH motif, which also demonstrates its high performance with *a* > 0.94 accuracy and *a* > 0.98 AUC.

We then evaluate the performance of RedNano and other methods at the transcriptome level on all three datasets. As shown in Supplementary Table S4, RedNano outperforms both EpiNano and nanom6A on the synthesized RNA dataset. Notably, RedNano accurately identifies the modification statuses of all RRACH sites with 100% accuracy, while EpiNano and nanom6A achieve accuracies of 0.9466 and 0.9808, respectively. It is worth noting that the high performance of these three methods is also due to the high read coverage of this dataset (mean coverage: >5057). On the *Arabidopsis* dataset, RedNano significantly outperforms DENA in detecting m6A in the RRACH motif, in terms of AUC (0.9405 versus 0.8763), AUPR (0.9474 versus 0.8941), and accuracy (0.8629 versus 0.6382) as shown in Fig. 2B and Supplementary Table S4. On the human dataset, RedNano also achieves higher AUCs and AUPRs than m6Anet (e.g. 0.8511 versus 0.8230 for RRACH and 0.8513 versus 0.8187 for DRACH in terms of AUC). We then use the results of GLORI (Liu *et al.* 2023) on the human HEK293T sample, which contains 145 294 DRACH m6A sites and 125 758 RRACH m6A sites, as a benchmark to evaluate RedNano. As shown in Supplementary Table S5, RedNano also outperforms m6Anet in terms of AUCs and AUPRs, further demonstrating the ability of RedNano to detect RNA m6A sites.

### 3.2 Cross-species evaluation of RedNano

**Figure 3.**
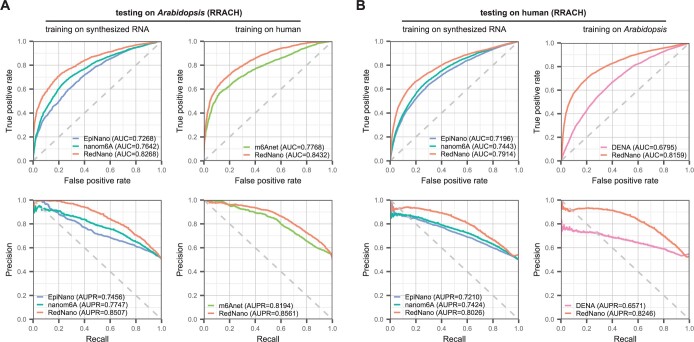
Evaluation of the generalizability of RedNano and other methods across two different species. (A) Testing on the *Arabidopsis* dataset. (B) Testing on the human dataset.

The ability of supervised methods to generalize is crucial for practical use, given that not all species have labeled datasets available for training. To evaluate the generalizability of the model of RedNano to data from different species, we conducted evaluations on the human and *Arabidopsis* datasets using models trained on datasets of different species. In tests on the *Arabidopsis* dataset, RedNano achieves higher performances than other methods. Specifically, when trained using the synthesized RNA dataset, RedNano surpasses EpiNano and nanom6A in terms of RedNano both AUC and AUPR. When trained using the human dataset, achieves an AUC of 0.8432 and an AUPR of 0.8561, which are >6% and >3% higher than those of m6Anet, respectively (Fig. 3A and Supplementary Table S4).

In tests on the human dataset, RedNano also achieves the highest performances, regardless of whether the models are trained using the synthesized RNA dataset or the *Arabidopsis* dataset (Fig. 3B and Supplementary Table S4). When trained using the synthesized dataset, RedNano achieves >4% higher AUC and >6% higher AUPR than nanom6A and EpiNano. When trained using the *Arabidopsis* dataset, RedNano achieves an AUC of 0.8159 and an AUPR of 0.8246, which is more than 13% and 16% higher than those of DENA, respectively. These results suggest that RedNano exhibits cross-species generalizability, and the per-trained models of RedNano are robust to new species.

We also explore how to use the training data from multiple datasets to improve the performance of RedNano. We combine the training data of multiple datasets to train a new RedNano model using the multiple-instance learning approach. Compared to the models trained using data from a single species, the new RedNano model gets the best overall performance across all datasets (Supplementary Note S4 and Fig. S4).

### 3.3 Evaluation of RedNano using the *P. trichocarpa* data

**Figure 4.**
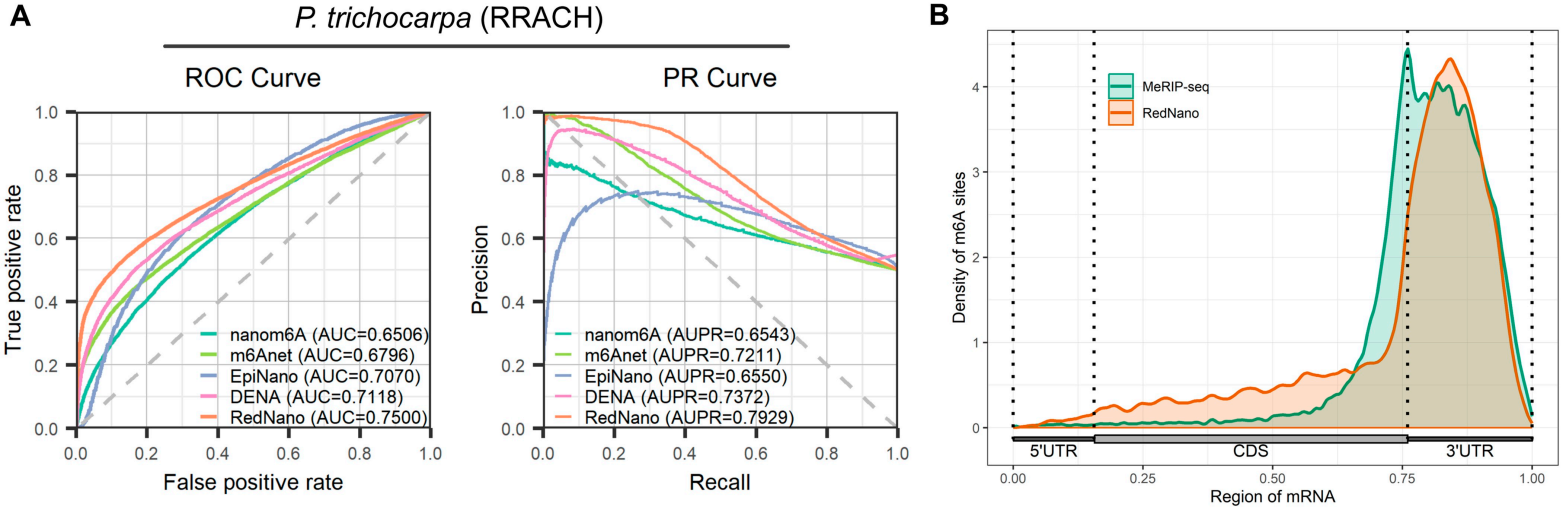
Evaluation on the *P.trichocarpa* dataset. (A) Performances of different methods for detecting m6A from the *P.trichocarpa* dataset. (B) The distribution of m6A sites on the transcripts.

To further assess the effectiveness of RedNano, we use a DRS dataset of *P.trichocarpa* which is not involved in training for evaluation. This *P.trichocarpa* dataset was obtained from Gao *et al.* (2021). We also got corresponding MeRIP-seq data from Gao *et al.* (2021) for orthogonal validation (Supplementary Note S1). We use the RedNano model trained on the combined dataset for prediction and compare its performance with nanom6A, m6Anet, EpiNano, and DENA. As shown in Fig. 4A, RedNano achieves the highest performance in detecting m6A sites in the RRACH motif of *P.trichocarpa* with an AUC of 0.7500 and an AUPR of 0.7929, which are 3.8%–9.9% and 5.5%–13.8% higher than other methods, respectively. The m6A sites predicted by RedNano are strongly enriched in the 3′-UTR region and around the stop codon, which is consistent with MeRIP-seq (Fig. 4B). These results demonstrate that RedNano with its pre-trained models is effective and robust in detecting m6A sites in new species and datasets.

### 3.4 Contribution of the raw-signal and basecalling-error features

In RedNano, we utilize both the raw signals and the basecalling errors as features for RNA m6A detection. The model architecture and hyperparameters of RedNano to process the two types of features are determined based on an evaluation of the human dataset, which are provided in Supplementary Fig. S5 and Supplementary Tables S7 and S8. To assess the contribution of each feature type, we further conduct an ablation experiment using the synthesized RNA, *Arabidopsis*, and human datasets. In this ablation experiment, we train the RedNano model using either the raw-signal feature or the basecalling-error feature with corresponding neural networks (Fig. 1). We train and test the models of RedNano using each of these three datasets independently. We focus on detecting m6A sites in only the RRACH motif in this experiment.

Supplementary Table S9 shows the results of the ablation experiment using the synthesized RNA dataset. RedNano achieves similar performances with accuracies of 0.9107 and 0.9073 when using the basecalling-error feature and the raw-signal feature alone, respectively. When both features are used, the performance of RedNano improves by over 4% in accuracy compared to using a single type of feature. The results on the *Arabidopsis* and human datasets also show that RedNano achieves the best performance when using both features (Table 1), while the accuracies, AUCs, and AUPRs of RedNano using a single feature are lower. Overall, these results indicate that both features contribute to RNA m6A prediction in different species and complement each other.

**Table 1.** Transcriptome-level evaluation of RedNano in the ablation experiment using the *Arabidopsis* and human datasets.^a^

Dataset	Feature	ACC	SEN	SPE	AUC	AUPR
*Arabidopsis*	BE	0.8130	0.7827	0.8437	0.8941	0.9064
	RS	0.8301	0.8177	0.8427	0.9112	0.9166
	BE+RS	0.8629	0.8191	0.9076	0.9405	0.9474
Humans	BE	0.7716	0.7041	0.8390	0.8454	0.8484
	RS	0.7546	0.7506	0.7585	0.8342	0.8347
	BE+RS	0.7735	0.7721	0.7749	0.8511	0.8519

a BE, basecalling-error feature; RS, raw-signal feature.

## 4 Discussion

Previous methods for m6A detection from Nanopore DRS reads typically use either statistical features of the current signals or basecalling-error features. In this study, we propose RedNano, a deep-learning method that utilizes two types of features, the raw signals and basecalling errors, to detect m6A from DRS reads. In RedNano, the raw-signal and basecalling-error features are processed by residual networks. Our experiments demonstrate that both these two types of features contribute to m6A prediction and complement each other. Testing on the synthesized RNA, *Arabidopsis*, and human datasets shows that RedNano is accurate and robust for RNA m6A detection at both the read level and transcriptome level across different species. Moreover, we found that by combining the samples of the three datasets for training, the RedNano model gets a more robust performance compared to the models trained on individual datasets. The effectiveness and robustness of RedNano and its pre-trained models for RNA m6A detection are further validated by an independent dataset of *P.trichocarpa*.

The ONT DRS technology offers a promising approach for the detection of RNA m6A and other types of modifications. However, of the current known 170 RNA modifications, only a few [such as pseudouridine (Begik *et al.* 2021) and inosine (Nguyen *et al.* 2022)] have been proven detectable by ONT DRS. One of our future works is to apply RedNano to detect more types of RNA modifications by establishing standard datasets for training and testing. At the same time, exploring the simultaneous detection of multiple types of RNA modifications using ONT DRS is also a promising research direction (Mateos *et al.* 2024). Additionally, The DRS technology is actively being developed by Oxford Nanopore Technologies. Recently, ONT released a new kit, RNA004, which is reported to significantly improve basecalling accuracy. As the technology continues to evolve, future work will also involve enhancing RedNano to adapt to the new DRS kits and data.

## Supplementary Material

btae375_Supplementary_Data

## Data Availability

All sequencing data used in this study is publicly available. The ONT DRS reads of the synthesized RNA can be accessed in National Center for Biotechnology Information (NCBI) under accession PRJNA511582. The DRS reads of the *Arabidopsis* vir-1 and VIRc lines can be accessed in European Nucleotide Archive (ENA) under accession PRJEB32782. The DRS reads of the human wild-type HEK293T cells can be accessed in ENA under accession PRJEB40872. The DRS reads of *P. trichocarpa* can be accessed in NCBI under the SRA accessions SRR8491764 and SRR1267667527.
