# Supplementary material for: RNA m6A detection using raw current signals and basecalling errors from Nanopore direct RNA sequencing reads
Source: Bioinformatics. 2024 Jun 18;40(6):btae375. doi: 10.1093/bioinformatics/btae375 (PMC11211211; doi:10.1093/bioinformatics/btae375)
Supplement: btae375_Supplementary_Data [file btae375_supplementary_data.pdf]

Supplementary Information for

RNA m6A detection using raw current signals and basecalling errors from Nanopore direct RNA sequencing reads

Supplementary Figures

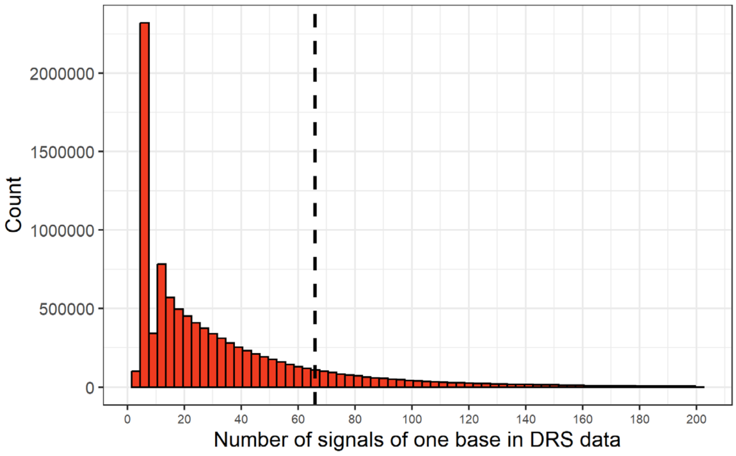

Fig. S1 Number of signals of 1 million randomly selected bases from the synthesized DRS data. Suppose u and σ are mean and standard deviation of the number of signals, the dashed line indicates approximately $u+\sigma$ (≈65).

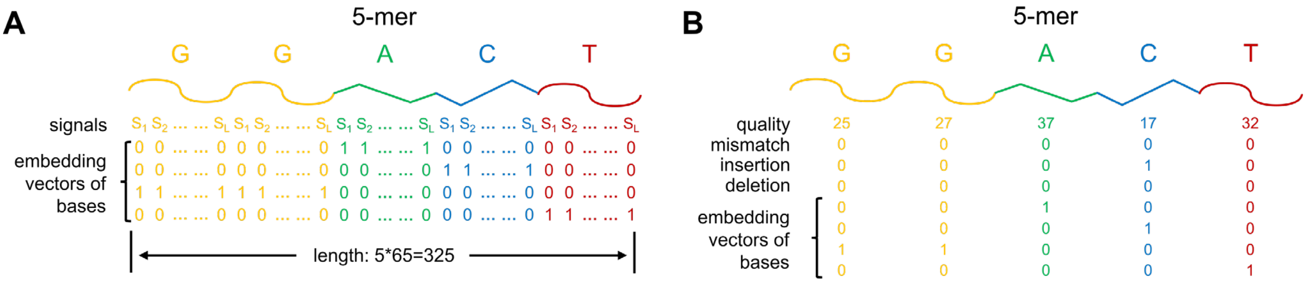

Fig. S2 Illustration of the raw-signal feature (A) and the basecalling-error feature (B) used in RedNano for a targeted site in a read.

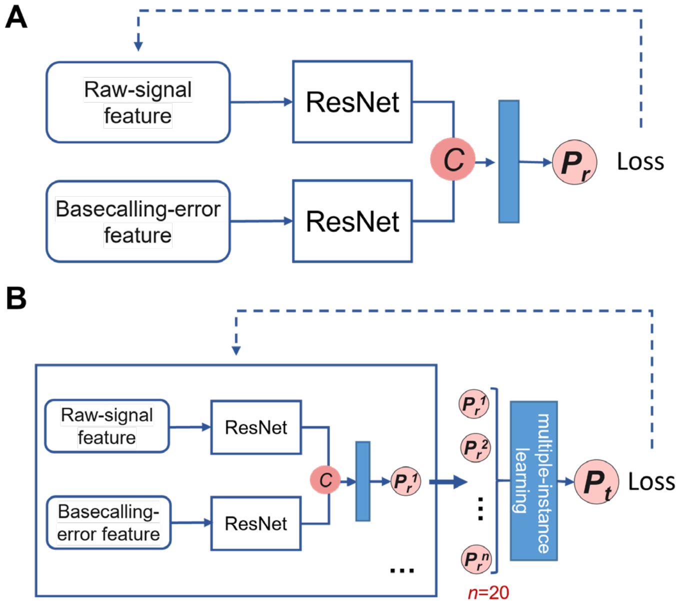

Fig. S3 Training approaches of RedNano. **A** Training at read level. **B** Training at transcriptome level using multiple instance learning. P_r is the read-level methylation probability, P_t is the transcriptome-level methylation probability.

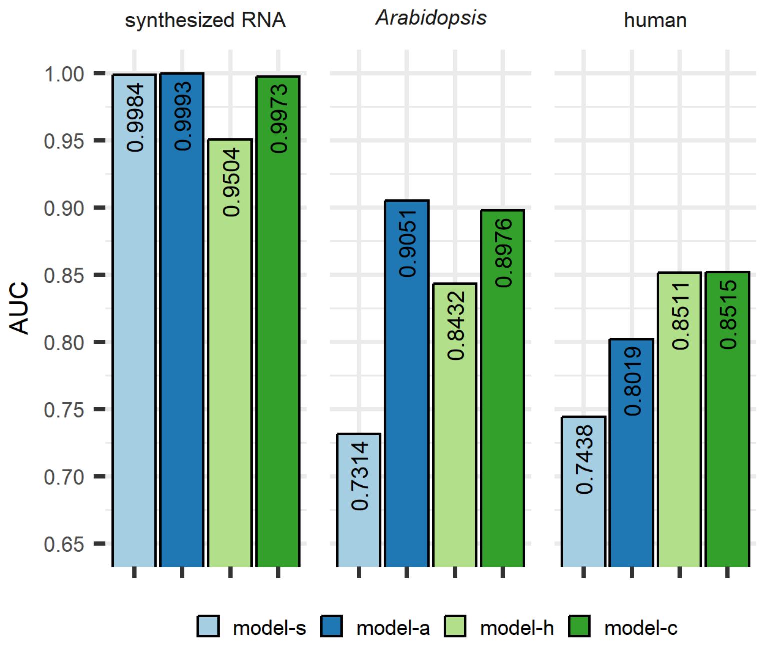

Fig. S4 Performance comparison of the RedNano models trained using different datasets.

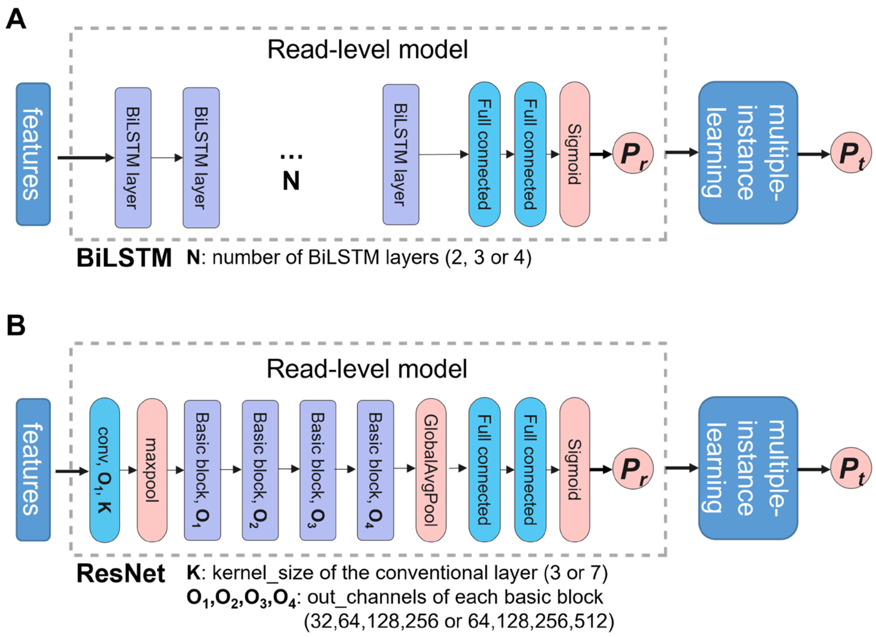

Fig. S5 Model architectures evaluated to process the input features. **A** Model used BiLSTM layers. **B** Model used ResNet. P_r is the read-level methylation probability, P_t is the trascriptome-level methylation probability.

Supplementary Tables

Table S1 Number of reads and the mean read coverage in each Nanopore DRS dataset used in this study. Numbers in table are in *NO. reads (mean coverage)* format.

**synthesized RNA**[1] (m6A motif: A)			
partition	replicate	modified	non-modified
training	rep1	638,860 (34,773.1)	846,595 (80,591.3)
testing	rep2	134,374 (6,218.8)	66,736 (5,057.3)
Arabidopsis[2] (m6A motif: RRACH)			
partition	replicate	vir1	VIRc
training	rep1	858,747 (13.2)	1,699,123 (23.5)
	rep2	1,842,868 (25.2)	1,678,723 (23.6)
testing	rep3	1,123,405 (16.0)	1,168,209 (18.0)
**human HEK293T**[3] (m6A motif: DRACH)			
partition	replicate	wild-type	
training	rep1	1,396,000 (11.8)	
	rep2	513,670 (5.6)	
testing	rep3	1,040,661 (8.7)	
P. trichocarpa[4] (m6A motif: RRACH)			
partition	replicate	wild-type	
testing	-	1,489,277 (24.0)	

Table S2 The targeted motifs, training data and prediction levels of four compared methods.

method	targeted motif	training data	prediction level
EpiNano[1]	RRACH	synthesized RNA	transcriptome-level
nanom6A[4]	RRACH	synthesized RNA	read-level, transcriptome-level
DENA[5]	RRACH	*Arabidopsis*	transcriptome-level
m6anet[6]	DRACH	human HEK293T	transcriptome-level

Table S3 Performances of RedNano on detecting RRACH m6A sites at transcriptome level from the human, Arabidopsis, and synthesized RNA datasets. RedNano-tl and RedNano-rl represents the multiple instance learning approach and the frequency calculation approach, respectively, to get the transcriptome-level methylation probability.

training dataset	method	testing dataset					
		synthesized RNA		*Arabidopsis*		human	
		AUC	AUPR	AUC	AUPR	AUC	AUPR
Synthesized RNA	RedNano-tl	0.9984	0.9984	0.7314	0.7586	0.7438	0.7666
	RedNano-rl	1.0000	1.0000	0.8268	0.8507	0.7914	0.8026
Arabidopsis	RedNano-tl	0.9993	0.9994	0.9051	0.9131	0.8019	0.8132
	RedNano-rl	0.9995	0.9995	0.9426	0.9517	0.8159	0.8246
human	RedNano-tl	0.9504	0.9580	0.8432	0.8561	0.8511	0.8519
	RedNano-rl	0.9314	0.9378	0.7536	0.7926	0.8390	0.8334

Table S4 Performance comparison of RedNano and other methods on detecting RNA m6As at transcriptome level using three datasets.

dataset (motif)	method	ACC	SEN	SPE	AUC	AUPR
synthesized RNA (RRACH)	EpiNano	0.9466	0.9542	0.9389	0.9947	0.9948
	nanom6A	0.9808	0.9615	1.0000	1.0000	1.0000
	RedNano	1.0000	1.0000	1.0000	1.0000	1.0000
Arabidopsis (RRACH)	DENA	0.6382	0.9764	0.2830	0.8763	0.8941
	RedNano	0.8629	0.8191	0.9076	0.9405	0.9474
human (RRACH)	m6Anet	0.7546	0.6758	0.8335	0.8230	0.8335
	RedNano	0.7735	0.7721	0.7749	0.8511	0.8519
human (DRACH)	m6Anet	0.7494	0.6454	0.8534	0.8187	0.8347
	RedNano	0.7761	0.7388	0.8133	0.8513	0.8571

Table S5 Performances of RedNano and m6Anet on detecting RNA m6As at transcriptome level from human HEK293T when using GLORI as benchmark.

motif	method	ACC	SEN	SPE	AUC	AUPR
DRACH	m6Anet	0.8391	0.7847	0.8935	0.9185	0.9259
	RedNano	0.8559	0.8501	0.8616	0.9292	0.9349
RRACH	m6Anet	0.8473	0.8038	0.8908	0.9260	0.9316
	RedNano	0.8619	0.8640	0.8598	0.9351	0.9395

Table S6 Cross-species evaluation of RedNano and other methods on detecting RNA m6A at transcriptome level using three datasets.

testing dataset (motif)	training dataset	method	ACC	SEN	SPE	AUC	AUPR
Arabidopsis (RRACH)	synthesized RNA	EpiNano	0.6524	0.7753	0.5274	0.7268	0.7456
		nanom6A	0.7039	0.6282	0.7820	0.7642	0.7747
		RedNano	0.7319	0.5490	0.9180	0.8268	0.8507
	human	m6Anet	0.6994	0.7302	0.6651	0.7768	0.8194
		RedNano	0.7196	0.8831	0.5532	0.8432	0.8561
human (RRACH)	synthesized RNA	EpiNano	0.6019	0.2617	0.9421	0.7196	0.7210
		nanom6A	0.6414	0.3728	0.9100	0.7443	0.7424
		RedNano	0.6405	0.3123	0.9686	0.7914	0.8026
	Arabidopsis	DENA	0.6364	0.6420	0.6308	0.6795	0.6571
		RedNano	0.6653	0.3608	0.9697	0.8159	0.8246

Table S7 Evaluation of different model architectures and fixed signal lengths for the raw-signal feature at transcriptome level.

model architecture	signal length	#parameters	AUC	AUPR
BiLSTM (2 layers)	65	566,529	0.8263	0.8263
BiLSTM (3 layers)	65	961,793	0.8325	0.8312
BiLSTM (4 layers)	65	1,357,057	0.8352	0.8328
ResNet (kernel_size=7, out_channels=(64,128,256,512))	65	3,914,497	0.8239	0.8250
ResNet (kernel_size=3, out_channels=(32,64,128,256))	65	998,273	0.8216	0.8206
ResNet (kernel_size=7, out_channels=(32,64,128,256))	65	998,913	0.8342	0.8347
ResNet (kernel_size=7, out_channels=(32,64,128,256))	55	998,913	0.8339	0.8332
ResNet (kernel_size=7, out_channels=(32,64,128,256))	45	998,913	0.8301	0.8299

Table S8 Evaluation of different model architectures for the basecalling-error feature at transcriptome level.

model architecture	#parameters	AUC	AUPR
ResNet (kernal_size=7, out_channels=(64,128,256,512))	3,915,857	0.8427	0.8463
ResNet (kernal_size=7, out_channels=(32,64,128,256))	999,601	0.8429	0.8463
ResNet (kernal_size=3, out_channels=(32,64,128,256))	998,577	0.8454	0.8484

Table S9 Read-level evaluation of RedNano in the ablation experiment using the synthesized RNA dataset. BE: basecalling-error feature; RS: raw-signal feature.

feature	ACC	SEN	SPE	AUC	AUPR
BE	0.9107	0.951	0.8705	0.9715	0.9685
RS	0.9073	0.9367	0.8779	0.967	0.9663
BE+RS	0.9545	0.9705	0.9386	0.9908	0.9906

Supplementary Notes

Supplementary Note 1 Processing the MeRIP-seq data of *P. trichocarpa*

We got the MeRIP-seq data of *P. trichocarpa* from Gao et al.[4] as the benchmark to evaluate the ONT DRS based methods. We use the a Nextflow pipeline called MeRIPseqPipe[7] to process the MeRIP-seq data. Raw MeRIP-Seq reads are first processed by fastp[8] (v0.19.7) for quality control. Then the high-quality reads are aligned to the reference genome using STAR[9] (v2.6.1b). Duplication reads are removed by Picard (v2.21.6, https://github.com/broadinstitute/picard). Finally, 14,203 m6A peaks are called using MACS2[10] (v2.1.2). And 207,802 m6A sites at single-base resolution are called using MATK[7] (v1.0). To enhance the reliability of the m6A benchmark, we retain only those RRACH m6A sites called by MATK that are within the peaks called by MACS2. This process results in a final set of 122,075 RRACH m6A sites. Any RRACH sites that are neither within the MACS2-called peaks nor among the MATK-called sites are considered as unmodified sites.

Supplementary Note 2 Commands used for data preprocessing

We use Guppy[11] (v3.1.5) to basecall Nanopore reads in FAST5 format as follows:

```
guppy_basecaller -i /path/to/fast5_input/ -r -s /path/to/fast5_output \
  --fast5_out -c rna_r9.4.1_70bps_hac.cfg --gpu_runners_per_device 2 \
  --chunks_per_runner 2500 --device CUDA:0
cat /path/to/fast5_output/*.fastq > test.fastq
```

To generate the raw-signal features, we use Tombo[12] (v1.5.1) to resquiggle the raw signals to reference transcriptome as follows:

```
tombo resquiggle --overwrite /path/to/fast5_output/workspace/ \
  /path/to/reference.transcriptome.fa \
  --fit-global-scale --include-event-stdev \
  --corrected-group RawGenomeCorrected_001 \
  --processes 30
```

To generate the basecalling-error features, we use minimap2[13] (v2.17), Samtools[14] (version 1.7), and the *sam2tsv* module of jvarkit[15] as follows:

```
minimap2 -t 30 -ax map-ont /path/to/reference.transcriptome.fa test.fastq | \
  samtools view -hSb | samtools sort -@ 30 -o test.bam
samtools index test.bam
samtools view -h -F 3844 test.bam | \
  java -jar /path/to/sam2tsv.jar -r /path/to/reference.transcriptome.fa > test.tsv
```

Supplementary Note 3 Model training and testing

RedNano utilizes two approaches to calculate the transcriptome-level methylation probability P_t (Fig. 1). When using the frequency calculation approach, the model of RedNano is trained at read level (Fig. S3A). When using the multiple-instance learning approach, the model of RedNano is trained directly at transcriptome level (Fig. S3B). Druing training using the multiple instance learning approach, we sample 20 reads to calculating P_t of each site. We extract training samples from the dataset used for training (Supplementary Table S1), and split the training samples (*i.e.*, features with corresponding labels) into the training and validation dataset at a ratio of 3:1. During model training, we train the model parameters by minimizing the binary cross-entropy loss. The binary cross entropy loss function calculates the loss as follows:

$$L(\theta) = -\frac{1}{N}\sum_{i=1}^{N}(y_i log(\hat{y}_i) + (1 - y_i)log(1 - \hat{y}_i)) \quad (1)$$

where $\hat{y}_i$ is the ith predicted value in the model output, y_i is the corresponding ground-truth label, N is the batch size (N=512 by default), and θ represents all trainable parameters during the model training. We employ the Adam[16] optimizer to train the model parameters, starting with an initial learning rate of 0.001. The learning rate is decayed by a factor of 0.1 after every two epochs. To prevent overfitting, we implement a dropout strategy in both the BiLSTM and ResNet neural networks, setting the dropout rate at 0.5. Additionally, we utilize an early stopping strategy. This involves monitoring the loss of the validation dataset during training. If the loss fails to decrease for five consecutive epochs, we stop the training and save the model parameters from the epoch with the lowest loss.

We evaluate our model at read level (using only the Syntheized RNA dataset) and at transcriptome level (using all three datasets) (Supplementary Table S1). Note that to get the transcriptome-level methylation probability P_t during testing, we use all the read-level methylation probability P_r of a site when using the frequency calculation approach. When using the multiple-instance learning approach, we sample 20 reads of each site to get a probability, and run the model 1000 times to get the mean probability value of the 1000 runs as the final P_t. Based on the methylation probabilities and the ground truth, the Receiver Operating Characteristic (ROC) Curve with the Area Under the ROC Curve (AUC), and the Precision-Recall Curve with the Area Under the PR Curve (AUPR) are used to measure the performances. We also use accuracy (ACC), sensitivity (SEN) and specificity (SPE) for evaluation as follows:

$$ACC = \frac{TP+TN}{TP+TN+FP+FN} \quad (2)$$

$$SEN = \frac{TP}{TP+FN} \quad (3)$$

$$SPE = \frac{TN}{TN+FP} \quad (4)$$

where TP, FP, TN, and FN represent true positives, false positives, true negatives, and false negatives, respectively. All experiments of RedNano and other methods were performed on a server with 48 CPU processors (Intel(R) Xeon(R) CPU E5-2630 v4 @ 2.20GHz), 4*12GB TITAN V GPUs, and 256GB RAM.

Supplementary Note 4 Combining training data from multiple datasets improves the overall performance of the RedNano model

We explore how to use the training data from multiple datasets to improve the performance of RedNano. By combining the training data of the synthesized RNA, *Arabidopsis*, and human, we train a model of RedNano with the multiple-instance learning approach for transcriptome-level probability prediction.

We refer to the RedNano model trained on the combined dataset as model-c, while those trained independently on synthesized RNA, *Arabidopsis*, and human samples are referred to as model-s, model-a, and model-h respectively. As shown in Fig. S4, model-c gets the best overall performance across all datasets, whereas the models trained using data from a single species show significant variations among different datasets. The results suggest that although RedNano is more roubst than other methods, the RedNano models trained using a single dataset are still biased to the species of the training dataset. By combining training data from multiple datasets, the RedNano model can learn features common to multiple species, thereby enhancing its overall performance.

Supplementary References

1 Liu, H. *et al.* Accurate detection of m6A RNA modifications in native RNA sequences. *Nature Communications* **10**, 4079, doi:10.1038/s41467-019-11713-9 (2019).

2 Parker, M. T. *et al.* Nanopore direct RNA sequencing maps the complexity of Arabidopsis mRNA processing and m6A modification. *Elife* **9**, e49658 (2020).

3 Pratanwanich, P. N. *et al.* Identification of differential RNA modifications from nanopore direct RNA sequencing with xPore. *Nature Biotechnology* **39**, 1394-1402, doi:10.1038/s41587-021-00949-w (2021).

4 Gao, Y. *et al.* Quantitative profiling of N6-methyladenosine at single-base resolution in stem-differentiating xylem of Populus trichocarpa using Nanopore direct RNA sequencing. *Genome Biology* **22**, 22, doi:10.1186/s13059-020-02241-7 (2021).

5 Qin, H. *et al.* DENA: training an authentic neural network model using Nanopore sequencing data of Arabidopsis transcripts for detection and quantification of N6-methyladenosine on RNA. *Genome Biology* **23**, 1-23 (2022).

6 Hendra, C. *et al.* Detection of m6A from direct RNA sequencing using a multiple instance learning framework. *Nature Methods* **19**, 1590-1598, doi:10.1038/s41592-022-01666-1 (2022).

7 Bao, X. *et al.* MeRIPseqPipe: an integrated analysis pipeline for MeRIP-seq data based on Nextflow. *Bioinformatics* **38**, 2054-2056 (2022).

8 Chen, S., Zhou, Y., Chen, Y. & Gu, J. fastp: an ultra-fast all-in-one FASTQ preprocessor. *Bioinformatics* **34**, i884-i890, doi:10.1093/bioinformatics/bty560 (2018).

9 Dobin, A. *et al.* STAR: ultrafast universal RNA-seq aligner. *Bioinformatics* **29**, 15-21, doi:10.1093/bioinformatics/bts635 (2012).

10 Zhang, Y. *et al.* Model-based Analysis of ChIP-Seq (MACS). *Genome Biology* **9**, R137, doi:10.1186/gb-2008-9-9-r137 (2008).

11 Wick, R. R., Judd, L. M. & Holt, K. E. Performance of neural network basecalling tools for Oxford Nanopore sequencing. *Genome Biology* **20**, 129, doi:10.1186/s13059-019-1727-y (2019).

12 Stoiber, M. *et al.* De novo identification of DNA modifications enabled by genome-guided nanopore signal processing. *bioRxiv*, 094672 (2017).

13 Li, H. Minimap2: pairwise alignment for nucleotide sequences. *Bioinformatics* **34**, 3094-3100, doi:10.1093/bioinformatics/bty191 (2018).

14 Li, H. *et al.* The sequence alignment/map format and SAMtools. *Bioinformatics* **25**, 2078-2079 (2009).

15 Lindenbaum, P. *Jvarkit: java utilities for bioinformatics*, <https://github.com/lindenb/jvarkit> (2015).

16 Kingma, D. P. & Ba, J. Adam: A method for stochastic optimization. *arXiv preprint arXiv:1412.6980* (2014).
